# Are “outsiders” in? Exploring the impact of outsourced workers’ perceived insider status and job value status on job performance

**DOI:** 10.3389/fpsyg.2023.1159022

**Published:** 2023-08-09

**Authors:** Jean Fan Yang, Wei Shi, Erica Wen Chen, Ben Nanfeng Luo, Jenny Zejun Zhao, Zhechen Yin, Jiaqi Tao

**Affiliations:** ^1^School of Labor and Human Resources, Renmin University of China, Beijing, China; ^2^Department of Management, The College of Business, Tallahassee, FL, United States

**Keywords:** flexible employment, outsourcing, perceived insider status, job value status, job performance

## Abstract

**Introduction:**

Outsourcing, one of the nonstandard employment forms, has been increasingly popular with a wide variety of industries and employers. However, much less is known about its consequences at the employee level, especially relative to standard-employed colleagues. Drawing on social categorization theory and the human resource architecture model, the study was to investigate how outsourced (vs. standard) employment form impacts employees’ perceived insider status and then job performance, as well as the moderating role of job value status.

**Methods:**

To examine these effects, we collected two-wave and multi-source questionnaires from a sample of 147 outsourced employees, 279 standard employees, and their immediate supervisors. And interviews with 31 employees, their supervisors, and human resources personnel provided further support for our findings.

**Results:**

The results showed that relative to standard employees, outsourced employees were lower in perceived insider status and indirectly worse in job performance. Furthermore, both the comparative effects were stronger among core-status than peripheral-status employees.

**Discussion:**

Our study contributes to outsourcing and widely nonstandard employment literature, bringing the research focus from employers to outsourced employees’ psychological and behavioral consequences. Also, we extended literature on the human resource architecture, through a deeper investigation on the issue of employment form-job value status (mis)matching as well as its impacts on employees.

## 1. Introduction

Nonstandard workers have become a major part of the organizational workforce and have received widespread attention over the past few decades (De Cieri et al., 2022). Organizations have increasingly recognized that the nonstandard workforce can serve as an effective way to maintain or improve their competitiveness by reducing labor costs (Wang et al., 2016), enabling flexibility (Smith, 1997) and rapid expansion via large-scale hiring (Guest, 2004; He et al., 2021), and increasing knowledge creation (Ferdous et al., 2022). Recently, industries and scholars have also reported that nonstandard employment is rapidly growing owing to the COVID-19 pandemic (Kramer and Kramer, 2020; Gunn et al., 2022), driving the reasonable flow and optimal allocation of the labor force (Lansbury, 2021). However, despite the growing prevalence of the nonstandard workforce, existing organizational research has largely neglected this group (Ashford et al., 2007; Oyetunde et al., 2022), simply assuming and treating these employees’ organizational experiences and management practices the same as those of standard workers.

Among the various forms of nonstandard employment relationships, such as temporary, part-time, contingent, multi-party, and disguised employment (Polivka et al., 2000), outsourcing attracts particular attention from organizations (Houseman, 2001). Outsourced employees are hired by one organization (often known as an agency or contractor) but perform work in another organization (known as a client organization; Bidwell et al., 2013). Apart from its importance and ubiquitous presence in the workforce nowadays, the recent focus of outsourcing research has shifted from how outsourcing activities impact a firm’s performance, flexibility, and sustainability toward outsourced employees at the individual level, especially regarding whether and how outsourced employees differ from standard ones (Boswell et al., 2012; Austin-Egole and Iheriohanma, 2021).

However, direct evidence of outsourced employees’ work experience seems scarce and controversial. On the one hand, outsourced employees were found to suffer from a lack of job security (Seong et al., 2012), have difficulty blending in Belcourt (2006), and face a shortage of opportunities (Straughan and Tadai, 2018), resulting in both “second-class” citizenship and worse performance when compared to standard employees (Qian et al., 2020). On the other hand, recent studies challenge the status quo regarding the invariable disadvantages outsourced employees work under. These suggest that some outsourced employees exhibit a more positive psychological experience and behavioral downstream effects than their peers, such as flexibility (Guest, 2004; He et al., 2021) and lower quitting intention (Boswell et al., 2012). Therefore, outsourced employees are under disadvantaged work conditions due to the nature of nonstandard employment, but whether they have worse experiences and performance than standard workers requires a more nuanced theoretical understanding and empirical examination.

To advance the limited knowledge on outsourced and nonstandard employees at large, we aimed to first understand how outsourced workers’ self-categorizations of their relationship with their client organization influences their work experience. As social categorization theory suggests, individuals put themselves and others into categories to simplify the world around them (Tajfel and Turner, 1986). Stamper and Masterson (2002) further conceptualized a psychological bond as perceived insider status (PIS), depicting the extent to which one considers oneself accepted by a group. This in-group feeling fuels employees’ sense of belonging (Ashforth and Mael, 1989), generating job security (Masterson and Stamper, 2003) and work motivation (Johnson et al., 2010), and eventually improves their job performance (Chen and Aryee, 2007). Moreover, while a higher PIS at workplace is increasingly recognized as a powerful psychological mechanism toward better performance (e.g., Zhao and Liu, 2020), a high power distance orientation and collectivistic culture enhance individuals’ need for PIS, such that employees in such a cultural context strive to attach their membership to the organizations where they work (Zhan et al., 2019).

Evidence from the literature on nonstandard hiring also suggests that nonstandard employees are likely to view themselves as organizational outsiders or even second-class citizens due to their temporary employment status (Stamper and Masterson, 2002; Lapalme et al., 2009; Boswell et al., 2012). In the case of outsourced workers, the triangular relationship (employee-contractor-client) renders their administrative attachments with client organizations fragile. As per the status characteristics and social categorization theories, the “second-class” employment form and loose administrative attachments hinder outsourced workers from acquiring insider status markers. Instead, they generate outsider markers and develop a sense of belonging and self-classification to neither the contractor nor client organization (often described as the dual-commitment problem; Boswell et al., 2012). In the inevitable and continuous comparison between themselves and their standardly employed colleagues, outsourced workers’ outsider perception aggregates and affects their subsequent attitudes and behaviors in the workplace (Chen and Aryee, 2007; Ouyang et al., 2015; Zhan et al., 2019).

Moreover, we propose that the above relationship is not ubiquitous to all outsourced labor forces, but varies according to the relative value to the organization. As Lepak and Snell’s (1999) human resource architecture model suggests, organizations are motivated to internalize employment when the human capital of the employee is valuable and unique. While standard and outsourced workers are considered to make different contributions in realizing strategic goals, differential human resource management (HRM) practices (e.g., employment forms) are applied (Lepak and Snell, 2007). However, when an unsuitable employment relationship appears (e.g., when employees of high value to the organization are hired via contractors), this imbalance between employment form and job value is likely to engender uncomfortable dissonance, resulting in negative emotional experiences, reduced work motivation, and worse job performance among employees (Tsui et al., 1997).

Given the above-mentioned limited and inconsistent research findings on outsourced employees, we first conducted a two-stage multi-source study among employees and their direct supervisors from the shipbuilding industry in China and then substantiated the findings via an interview study with employees of both forms, their leaders, and human resources (HR) workers. We aimed to contribute to the literature in several ways. First, while the nonstandard literature has a long-standing observation that the nonstandard workforce often experiences disadvantages and performs worse than their regularly employed counterparts (e.g., Haines et al., 2018), direct empirical evidence on why it happens is still limited (with a few exceptions such as Boswell et al., 2012; Eldor and Cappelli, 2021). As such, a more sophisticated understanding of the phenomenon is needed (see Signoretti et al., 2022 for a recent call). By showing how outsourced and standard employees achieve differential performance via the extent to which they recognize themselves as organizational insiders, this study can increase our knowledge of the psychological mechanisms underlying the relation between nonstandard employees’ employment arrangements and work performance (Cappelli and Keller, 2013; Spreitzer et al., 2017; Spurk and Straub, 2020).

Second, by showing how job value status serves as a vital boundary condition, our work also contributes to the serial discussion on the employee-organization relationship (Tsui et al., 1997) and human resource architecture (Lepak and Snell, 1999; Marchington, 2015; Luo et al., 2021). In Lepak and Snell’s (2002) influential work, the strategic value and uniqueness of human capital are the major drivers of employment modes because an ideal human resource architecture should be a purposeful balance between employment modes and human capital. Job characteristics are further introduced to help categorize the strategic value, level of uniqueness, and matched employment modes of human capital. Therefore, different from most follow-up studies, which adopt this framework to identify an “ideal” employment mode for a differential workforce during a firm’s different life stages (e.g., Bryant and Allen, 2011; with Keegan and Meijerink, 2022 as an exception), our work is among the few to empirically investigate how workforce values and employment modes jointly influence employees’ psychological experience and behavioral outcomes.

Third, as the nonstandard workforce practice and HRM environment in China have been undergoing significant changes (Zhao et al., 2021), understanding why nonstandard workers perform differently and how the employment forms fit (or not) the job values is particularly timely and important. Rather than arbitrarily assuming that employees with the same contracts will homogenously think and behave similarly, we propose that employees with different values regarding their job should be hired with careful consideration of their cultural roots. Mismatches such as outsourced core workers and standard peripheral workers in a highly collectivistic culture could be detrimental to both individuals and firms.

In the sections that follow, we first provide an overview of outsourced employment and how it differs from other employment arrangements. Then, we propose a set of hypotheses based on our review of existing findings in the literature. Following this, we introduce the methodology used to examine our research model and empirical findings. Finally, we conclude our findings with recommendations for further research.

## 2. Theory and hypotheses

### 2.1. Outsourced employment

Outsourced employees are a typical type of nonstandard workers who are hired by labor agency companies but work in client organizations for a specific period. Their employment could be fixed-term, a project/task-based contract, or seasonal or casual work (International Labour Organization, 2016). In contrast to the traditional bilateral relationship between employees and employers, the triangular relationship outsourced employees experience creates divisions between employment and actual work. On the one hand, outsourced employees are directly hired and administratively managed by labor agency organizations. On the other hand, they are asked to perform their activities to satisfy the needs of client organizations that are often in charge of defining, controlling, and providing day-to-day supervision of those activities (Bonet et al., 2013).

In recent years, the outsourcing of workers has become increasingly popular among employers, as it may reduce costs and provide organizations more flexibility to cope with increasing global competition and uncertainty (Ashford et al., 2007; Bidwell et al., 2013; De Stefano et al., 2019). The development of temporary help agencies and contract companies that act as employment intermediaries (Kalleberg and Marsden, 2005) and the advent of inexpensive communication technologies and digitalization (Zhao et al., 2021) have greatly facilitated the use of outsourced workers (Bellace, 2018). As a result, more companies are hiring outsourced workers to replace fixed staff, making outsourcing employment more feasible and ubiquitous today.

Although the number and importance of outsourced employees has shown sustained growth, the empirical research on this group of employees is limited and controversial. Outsourcing is often included in the umbrella-term nonstandard employment research (Chadwick and Flinchbaugh, 2016), which reports inconsistent results regarding whether the use of flexible employees can lead to positive outcomes. For example, many studies have shown that nonstandard work relationships might plausibly be associated with overworking (Arlinghaus et al., 2019), high job and financial insecurities (Cooke, 2014; Rubery et al., 2016; Peticca-Harris et al., 2020), a sense of marginalization and social isolation (Hoque and Kirkpatrick, 2003), loss of opportunity for development (Peticca-Harris et al., 2020), low job crafting behaviors (Plomp et al., 2019), low organizational identification (Seong et al., 2012), discontinuous employment, and short-term career expectations (Peticca-Harris et al., 2020). However, some research challenges the conclusion that flexible workers are invariably disadvantaged, instead contending that flexible employment may provide more options and flexibility for workers (He et al., 2021), solve the short-term employment problem of some people, and achieve greater personal benefits (Green and Heywood, 2011). Some researchers hold that mixed results are partly attributable to the disparate nature of nonstandard work arrangements and tendency for studies to obscure differences between types of nonstandard work, such as the length of employee contracts and expectation of continued employment (Coyle-Shapiro et al., 2006; Hughes and Palmer, 2007). To mitigate the inconsistent results that can stem from treating nonstandard work arrangements homogeneously (Chadwick and Flinchbaugh, 2016), this study focuses on a particular type of nonstandard employment, namely outsourced employment.

### 2.2. The mediating effect of employees’ perceived insider status

Perceived insider status (PIS) depicts employees’ self-categorizations of the psychological relationship between themselves and their organizations (Stamper and Masterson, 2002; Chen and Aryee, 2007; Dai and Chen, 2015). A high level of PIS can generate various benefits (Boswell et al., 2012) such as a stronger sense of belonging (Schaubroeck et al., 2017), higher organizational commitment (Wang et al., 2021), higher satisfaction and retention (Armstrong-Stassen and Schlosser, 2011; Knapp et al., 2014; Chen et al., 2017), higher well-being in the workplace (Findler et al., 2007), less follower territorial behavior (Liu et al., 2021), and more organizational citizenship behaviors (Caron et al., 2019). Noteworthy is that we chose PIS rather than organizational identification because scholars have found PIS is particularly salient in the Chinese context (Hui et al., 2015) due to the highly collective and power distance-orientated culture. As cultural psychologists have found, Chinese employees view adhering to their social roles (Westwood et al., 2004) and gaining insider status as an important goal and are thus motivated to devote more effort to their job (Lu, 2008). This suggests its theoretical appropriateness for the current study.

A critical feature of PIS is that it depicts an individual’s perception of their relative standing in an organization (Zhan et al., 2019). As status characteristics theory (Berger et al., 1972) and social categorization theory (Tajfel and Turner, 1986) suggest, individuals use distinctive signs or indicators to identify different classes and then classify themselves as members of a collective group (Schaubroeck et al., 2017), which can inadvertently establish status distinctions. Employment arrangement serves as such a status characteristic whereby one type of arrangement may be perceived as having higher status than the other (Boswell et al., 2012). In the case of outsourced and standard employees, outsourced employees are hired and get paid by agency organizations, but perform work in client organizations, experiencing comparatively disadvantaged relationships with the client organization. In fact, temporary agencies sometimes treat outsourced employees as a set of skills rather than as individuals, which may aggravate their perception of an outsider status (Inkson et al., 2001). These two groups are likely to receive different signals such as rewards or incentives from client organizations (e.g., training, promotion opportunities, health plans, and social functions; Lapalme et al., 2009), and therefore have different status markers (Boswell et al., 2012). In other words, the differences in employment arrangements are salient, resulting in persistent status markers (Broschak and Davis-Blake, 2006), which are likely to lead standard workers to have insider status while outsourced workers have outsider status. Moreover, the relationship between outsourced employees and the organizations they work for is indeterminate and ambiguous, relegating outsourced employees to the out-group, which may subsequently be ascribed a lower status (Boswell et al., 2012).

When employees deem their status, for example, as more of an insider or outsider, they behave accordingly. Employees with a high PIS tend to be involved in the organization because of strong affective commitment (Lee and Peccei, 2007), and are willing to contribute intelligence and power to it (Caron et al., 2019), resulting in better overall job performance (Wang and Kim, 2013; Schaubroeck et al., 2017). In sum, considering status characteristics as well as self-classification and categorization, compared with standard employees, outsourced employees are more likely to experience lower levels of PIS and consequently, exhibit lower job performance. Thus, we hypothesized the following:

*H1*: PIS plays a mediating role between employment forms (standard vs. outsourced) and job performance. Compared to standard employees, outsourced employees are more likely to have lower PIS and job performance.

### 2.3. The moderating effect of employees’ job value status

Although outsourced employees are more likely to have lower PIS and job performance than standard employees, previous empirical research has suggested that their status perceptions do not always match the objective categories under which they are placed (Lapalme et al., 2009). In other words, even though outsourced employees are more likely to be classified as outsiders, some may consider themselves less as outsiders, and instead be similar to the organization’s typical insider standard employees.

To further explore the boundary conditions that shift employees’ PIS, we adopted the human resource architecture model and argue that the value of human capital is inherently dependent on its potential to contribute to an organization’s competitive advantage or core competence. Thus, we categorize employees’ job values as core or peripheral according to their relative value to the firm (Lepak and Snell, 1999, 2002). Core employees are vital to an organization’s competitive advantage, motivating it to acquire and develop these employees. By contrast, peripheral employees are suited for contracting or creating human capital alliances because their human capital is not unique or of strategic value to a firm (Collins et al., 2021).

As organizations should maintain long-term involvement and investment in core employees to facilitate their successes, the use of standard employment for core employees is expected and appropriate for both organizations and employees (Coyle-Shapiro and Kessler, 2002). However, if these core employees cannot obtain long-term standard employment, but are hired in the outsourced employment form, an imbalance (here often denoted as under-investment by organizations) emerges (Tsui et al., 1997). In other words, core employees are expected to undertake broad and open-ended obligations, while their employer reciprocates with temporary contracts, resulting in no commitment to a long-term relationship or investment in their employees’ training or careers (Jia et al., 2014). Therefore, core employees with a flexible form of employment will be less likely to make affective commitments to the organization they belong to, as there is no expectation of employment security and a desirable payoff. Consequently, outsourced core employees will have lower PIS in their organization than their standard core employee colleagues, and the differences in PIS and performance between the two groups will be augmented.

However, peripheral employees such as assembly line workers are often hired through outsourcing and other nonstandard employment forms (Coyle-Shapiro and Kessler, 2002). As their added value is low and their skills are generic, peripheral employees only need to perform well-specified aspects of job-focused activities (Shore et al., 2004). Employment decisions around peripheral employees often focus on how to reduce costs rather than increase human capital, and organizations only seek limited continuity and loyalty from this group (Kalleberg, 2003). Consequently, when organizations offer temporary contracts to peripheral employees via agency companies, a type of balance called quasi-spot contract emerges, which is a relatively short-term, closed-ended, and purely economic decision (Tsui et al., 1997). However, if these employees are provided long-term or even permanent contracts, a contrasting type of imbalance (here often denoted as over-investment by organizations) appears (Lengnick-Hall et al., 2009). In this case, peripheral employees perform low value-added activities but receive open-ended rewards. As a result, those peripheral employees hired in the form of standard employment will experience higher PIS compared to their outsourced employee colleagues as a result of feeling recognized and accepted by the organization. However, the situation of over-investment by organizations is similar to that of over-payment. Even though the organization’s investment is higher than it is in the quasi-spot contract, recipients tend to rationalize the over-payment; thus, the more favorable investment does not act as an incentive (Tsui et al., 1997). Therefore, the differences in PIS and performance between the two groups (outsourced vs. standard peripheral employees) will be undermined. Hence, we hypothesized the following:

*H2*: The mediated relationship between employment forms (standard vs. outsourcing) and job performance via PIS will be moderated by employees’ job value statuses (core vs. peripheral), such that the difference in PIS and performance between the two forms will be larger for core employees than for peripheral employees.

## 3. Methods

### 3.1. Sampling

We collected data from eight shipbuilding firms in China with flexible employment and labor intensity. China has been undergoing profound social, economic, and technological changes (Zhao et al., 2021) in which outsourcing employment has emerged. This form of employment has been widely used for many years, especially in traditional labor-intensive industries, and researchers have highlighted the dynamics of employment relations in Asian countries (Budhwar and Debrah, 2009). Most of our sample was employed in the production and technology departments, demonstrating high consistency and representativeness. To reduce the risk of common method variance, we adopted a multi-source and multi-wave research design by collecting data from both employees and their supervisors with a time interval of two weeks. We first communicated with the HR department of each firm about the purpose and design of our study, and asked for their assistance. Then, questionnaires that included questions regarding basic personal and job information and PIS were distributed to employees with the HR departments’ coordination. Finally, two weeks after employees’ responses were received, we asked their supervisors to complete a short survey evaluating their employees’ job performance. Both employees and their supervisors were informed that all survey data would be used for research purposes only, and their participation information, personal data, and questionnaire responses would be kept strictly confidential in accordance with academic and ethical guidelines.

From December 2019 to April 2020, we undertook a two-stage multi-source data collection. In the first stage, we distributed 920 questionnaires to employees in eight firms and collected 603 effective responses (response rate = 65.54%). After two weeks (stage two), we asked the supervisors of these 603 employees to evaluate their subordinates’ job performance, and 426 employee-supervisor-matched responses were collected (response rate = 70.65% for this second-round collection). Those 177 lessened responses at stage two included (a) 168 samples from the first stage that did not get supervisors’ ratings of employees’ job performance in stage two and (b) 9 samples that consisted of missing data and repeated strings that we commonly recognized as quality signals. The relative percentage was aligned with the employment profile of the Chinese shipbuilding industry. More than 96% of the sample was male, and the average age was more than 30 years. The average education level was below junior college or higher vocational school, and more than 60% indicated an organizational tenure of more than 5 years.

### 3.2. Measures

As mentioned, we collected information on employment forms, job value status, and PIS from employees, and employees’ job performance evaluations from their supervisors. The scales were originally constructed in English, and Brislin’s (1980) translation-back translation procedure to translate all items into Chinese was strictly followed. Before formally distributing the questionnaire, we sought feedback from five employees and two supervisors regarding the clarity of the questions, and adopted their advice where indicated. For the Likert-scale item responses, we used a five-point scale ranging from 1 = “Strongly disagree” to 5 = “Strongly agree.”

#### 3.2.1. Employment form

We measured the form of employment through a dummy variable, with 1 representing standard employees and 0 representing outsourced employees, in accordance with previous similar approach by Henkens and Leenders (2010), Boswell et al. (2012), and Ferguson et al. (2012).

#### 3.2.2. Job value status

In accordance with the literature on human resource architecture (e.g., Tsui et al., 1997), we used employees’ job type as a proxy for their job value status. We further examined archival information on each job design in these firms. We also conducted 11 interviews with the HR managers from each firm to classify the value of each job. Based on these data, we then generated a dummy variable from the employee’s job position to capture their job value status. Specifically, those working in academic research, R&D, or management were categorized as core employees and assigned a numerical value of 1, while those working in technical manipulation, technical service, mechanic work, or sales were categorized as peripheral employees and assigned a numerical value of 0. To validate the external validity of this measurement, we conducted a new data collection on an online data platform (Credamo) in China. Specifically, we asked respondents to evaluate the value of each job type in a manufacturing enterprise setting, using a Likert scale point of one to seven. One hundred twenty three questionnaire responses were returned for analysis. After assigning these seven job types into two groups as the dummy variable, the between-group comparison is statistically significant (difference = 1.61, *p* < 0.001). The mean score of core status workers’ job value is 6.22 (SD = 0.52), while the mean score of peripheral workers’ job value is 4.61 (SD = 1.03). The median score of core status workers’ job value is 6.33, while the median score of peripheral status workers’ job value is 4.75.

#### 3.2.3. Perceived insider status

We measured employees’ PIS using Stamper and Masterson’s (2002) five-item scale. Items included: “I feel very much a part of my work organization,” “My work organization makes me believe that I am included in it,” “I feel like I am an ‘outsider’ at this organization” (reverse item), “I do not feel included in this organization” (reverse item), and “My work organization makes me frequently feel ‘left out’” (reverse item). Higher scores represented higher levels of PIS. The Cronbach’s α of this scale in the current study was 0.93.

#### 3.2.4. Job performance

Employees’ job performance was evaluated by their supervisors using a five-point four-item scale from Van Dyne and LePine (1998). Items included: “This particular worker fulfills the responsibilities specified in his/her job description,” “This particular worker performs the tasks expected as part of the job,” “This particular worker meets performance expectations,” and “This particular worker adequately completes responsibilities.” Higher scores represented higher levels of job performance. The Cronbach’s α of this scale in the current study was 0.87.

#### 3.2.5. Control variables

Control variables included gender (0 = female, 1 = male), age, education level (0 = senior high school or below, 1 = technical secondary school or technical school, 2 = junior college or higher vocational school, 3 = bachelor’s degree, 4 = master’s degree or above; the categories are based on the current Chinese education system), and organizational tenure.

## 4. Results

### 4.1. Confirmatory factor analyses

Confirmatory factor analyses were first conducted to test the discriminant validity of the PIS and job performance measurements, as both employment form and job value status were dummy variables. The results indicated that the two-factor model had a good fit: *χ*^2^ = 96.34, df = 26, root mean square error of approximation (RMSEA) = 0.08, comparative fit index (CFI) = 0.98, Tucker-Lewis index (TLI) = 0.97, standardized root mean squared residual (SRMR) = 0.02. Further, the fit of the two-factor model was significantly better than that of the one-factor model: *χ*^2^ = 114.20, df = 27, RMSEA = 0.09, CFI = 0.97, TLI = 0.96, SRMR = 0.03 (∆*χ*^2^ = 17.86, ∆df = 1, *p* < 0.01). Despite the multi-source and multi-wave research design, following Podsakoff et al. (2003), we also added common method variance as a latent factor to the two-factor model, which did not show significant improvement: *χ*^2^ = 102.69, df = 25, RMSEA = 0.09, CFI = 0.98, TLI = 0.97. This helped us rule out the implications of common method bias in hypothesis testing.

### 4.2. Descriptive statistics

Table 1 shows the means, standard deviations, and correlation coefficients for all variables. The correlation results are aligned with those of previous related research and theory.

**Table 1:** Descriptive statistics and correlations.

Variables	Mean (M)	SD	1	2	3	4	5	6	7
Gender	0.96	0.19							
Age	4.29	0.92	0.24**						
Education	1.69	0.98	−0.32**	−0.20**					
Tenure	3.29	1.01	−0.05	0.39**	0.00				
Employment form	0.65	0.48	0.01	−0.09	0.01	−0.15**			
Workers’ job value status	0.43	0.50	−0.10*	−0.01	0.06	0.08	−0.05		
Perceived insider status	3.77	0.96	0.02	0.02	0.06	−0.04	0.77**	−0.20**	
Job performance	3.98	0.79	0.01	0.06	0.07	−0.02	0.71**	−0.18**	0.87**

*N* = 426 employees. SD, standard deviation. For employment form, 1 = standard, 0 = outsourced. For workers’ job value status, 1 = core-status, 0 = peripheral-status. *p < 0.05. **p < 0.01.

### 4.3. Hypothesis testing

Multiple regression analyses were conducted to examine both the direct and indirect effects of employment form on job performance. First, we hypothesized that PIS would play a mediating role in the relationship between employment form and job performance. As Table 2 shows, employment form (standard employees vs. outsourced employees) was positively related to PIS (Model 1: *b* = 1.55, SE = 0.06, *p* < 0.01). PIS was also positively related to job performance (Model 3: *b* = 0.21, SE = 0.06, *p* < 0.01), and the direct effect of employment form remained significant (*b* = 0.21, SE = 0.06, *p* < 0.01). Moreover, we used a Monte Carlo bootstrap simulation with 5,000 replications to create our bias-corrected 95% confidence intervals (CIs) around the indirect effect. The bootstrapping results showed that employment form had a positive effect on job performance *via* PIS (estimate = 0.99, SE = 0.09, 95% CI [0.83, 1.17]). These results indicate that outsourced employees had lower levels of PIS and job performance than standard employees. Thus, Hypothesis 1 was supported.

**Table 2:** Estimated coefficients of the mediation model.

	Model 1		Model 2		Model 3	
	Perceived insider status		Job performance		Job performance	
	OLS	Heckman	OLS	Heckman	OLS	Heckman
*Main*						
Gender	0.10	0.10	−0.02	−0.02	−0.08	−0.08
	(0.61)	(0.61)	(−0.11)	(−0.12)	(−0.77)	(−0.76)
Age	0.09*	0.09*	0.11***	0.11***	0.05*	0.05*
	(2.42)	(2.44)	(3.32)	(3.33)	(2.25)	(2.22)
Education	0.08*	0.08*	0.07*	0.07*	0.02	0.02
	(2.34)	(2.35)	(2.39)	(2.40)	(1.04)	(1.05)
Tenure	0.04	0.04	0.03	0.03	0.01	0.01
	(1.12)	(1.10)	(1.05)	(1.00)	(0.37)	(0.26)
Employment form	1.58***		1.21***		0.21***	
	(24.99)		(21.37)		(3.38)	
1. Employment form		1.60***		1.21***		0.19
		(8.32)		(6.33)		(0.89)
Perceived insider status					0.63***	0.63***
					(20.25)	(20.42)
_cons	2.00***	1.99***	2.52***	2.51***	1.25***	1.27***
	(8.50)	(7.00)	(11.92)	(9.46)	(7.71)	(5.27)
*Employment form*						
Gender		0.14		0.14		0.14
		(0.40)		(0.40)		(0.40)
Age		−0.05		−0.05		−0.05
		(−0.67)		(−0.67)		(−0.68)
Education		0.02		0.02		0.02
		(0.31)		(0.31)		(0.31)
Tenure		−0.18*		−0.18*		−0.18*
		(−2.48)		(−2.48)		(−2.48)
_cons		1.05*		1.05*		1.06*
		(2.18)		(2.18)		(2.18)
**/**						
athrho		−0.02		−0.01		0.04
		(−0.11)		(−0.04)		(0.11)
lnsigma		−0.50***		−0.61***		−0.95***
		(−14.50)		(−17.75)		(−27.14)
N	426	426	426	426	426	426
Adj. *R*-sq	0.60		0.52		0.76	
*pseudo R ~ q*						

*N* = 426 employees. SE, standard error. For employment form, 1 = standard, 0 = outsourced. t statistics in parentheses. *p < 0.05, **p < 0.01, ***p < 0.001.

**Figure 1:**
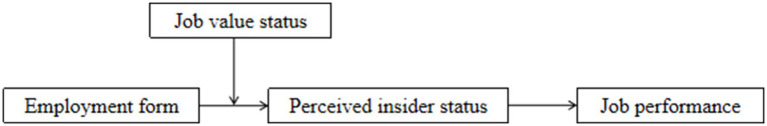
Theoretical model.

**Figure 2:**
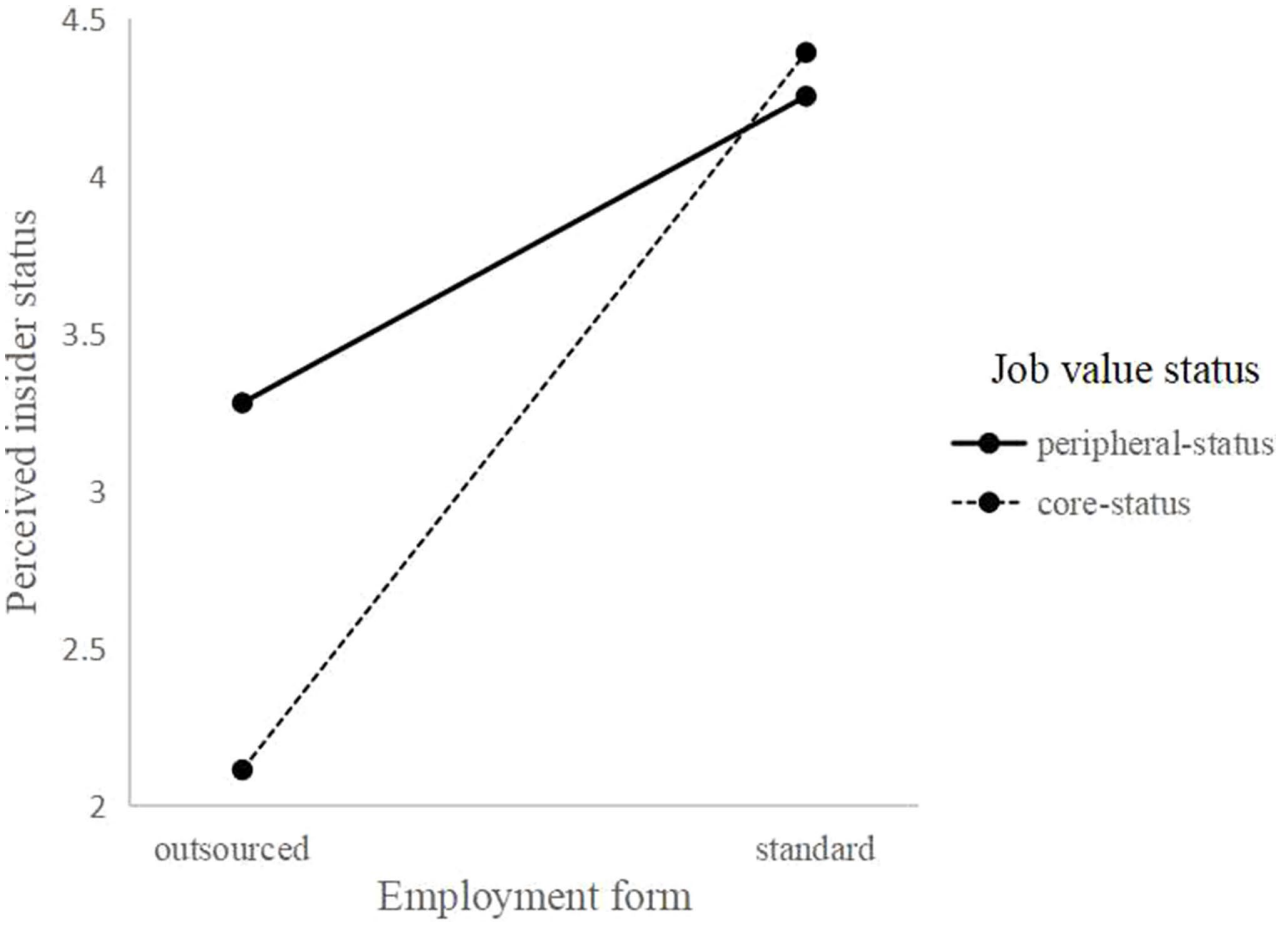
The moderating role of workers’ job value status on the relationship between employment forms and perceived insider status.

We also hypothesized that the indirect effect of employment form on job performance *via* PIS would be moderated by job value status. Table 3 shows the results of these analyses. In the first step, after the interaction term of employment form (standard employees vs. outsourced employees) and job value status (core employees vs. peripheral employees) were added to the regression model, the results showed that the interactive effect between employment form and job value status on PIS was significant and positive (Model 4: *b* = 1.30, SE = 0.11, *p* < 0.01). In the second step, we conducted a simple slope test, and as Figure 2 shows, employment form had a strengthened positive effect on PIS among core employees (*b* = 2.28, SE = 0.08, *p* < 0.01) more than among peripheral employees (*b* = 0.97, SE = 0.07, *p* < 0.01). In brief, the difference in PIS between core-standard and core-outsourced employees was greater than that between peripheral-standard and peripheral-outsourced employees.

**Table 3:** Estimated coefficients of the moderated mediation model.

	Model 4		Model 5	
	Perceived insider status		Job performance	
	OLS	Heckman	OLS	Heckman
*Main*				
Gender	0.05	0.02	−0.09	−0.04
	(0.38)	(0.12)	(−0.82)	(−0.33)
Age	0.06	0.06	0.05*	0.05*
	(1.92)	(1.95)	(2.26)	(1.98)
Education	0.06*	0.07*	0.02	0.02
	(2.42)	(2.54)	(1.19)	(0.68)
Tenure	0.03	0.03	0.01	0.01
	(1.17)	(1.25)	(0.45)	(0.25)
Employment form	0.97***	0.97***	0.20**	0.20**
	(13.83)	(13.87)	(3.04)	(3.08)
Workers’ job value status	−1.17***	−1.17***	−0.24**	−0.25**
	(−13.88)	(−13.96)	(−3.11)	(−3.18)
Employment forms × Workers’ job value status	1.30***		0.26**	
	(12.47)		(2.79)	
1. Employment forms × Workers’ job value status		1.05***		0.63**
		(3.58)		(3.28)
Perceived insider status			0.56***	0.56***
			(15.08)	(15.22)
_cons	2.76***	2.84***	1.54***	1.44***
	(13.67)	(12.85)	(8.30)	(7.23)
*Employment forms × Workers’ job value status*				
Gender		−0.36		−0.38
		(−1.04)		(−1.08)
Age		0.02		0.03
		(0.28)		(0.32)
Education		0.08		0.08
		(1.17)		(1.12)
Tenure		0.04		0.04
		(0.49)		(0.52)
_cons		−0.62		−0.62
		(−1.28)		(−1.26)
**/**				
athrho		0.30		−0.58*
		−0.91		(−2.14)
lnsigma		−0.67***		−0.88***
		(−10.58)		(−11.54)
*N*	426	426	426	426
Adj. *R*-sq	0.73		0.76	
*pseudo R ~ q*				

*N* = 426 employees. SE, standard error. For employment form, 1 = standard, 0 = outsourced. For workers’ job value status, 1 = core-status, 0 = peripheral-status. *t* statistics in parentheses.*p < 0.05, **p < 0.01, ***p < 0.001.

In the third step, the bootstrapping results (sample = 5,000) showed that job value status (core employees vs. peripheral employees) positively moderated the indirect effect of employment form (standard employees vs. outsourced employees) on job performance *via* PIS (estimate = 0.82, SE = 0.09, 95% CI [0.65, 1.02]). Specifically, the indirect effect was stronger among core employees (estimate = 1.44, SE = 0.11, 95% CI [1.22, 1.66]) than peripheral employees (estimate = 0.61, SE = 0.06, 95% CI [0.49, 0.74]). These results indicate that the difference in job performance *via* PIS between core-standard and core-outsourced employees was also greater than that between peripheral-standard and peripheral-outsourced employees. Thus, Hypothesis 2 was supported.

We also ran the Heckman two-stage model (Heckman, 1979, 1990) as a supplement to the OLS model. In the first stage, the probability of the self-selected variables (i.e., employment form and its interaction with workers’ job value status) valuing 1 was estimated. In the second stage, the estimated probability was added to the regression model to correct the self-selection bias. Our comparisons between the results of the Heckman two-stage model and the OLS model are also shown in Tables 2, 3 and the conclusion was unchanged.

In addition, we performed independent-sample t-tests to compare the PIS and job performance of the four employee types (core-standard, core-outsourced, peripheral-standard, peripheral-outsourced). Tables 4, 5 show the results for both PIS and job performance. Core-standard employees had the highest levels, while core-outsourced employees had the lowest, and peripheral-standard employees had higher levels than peripheral-outsourced employees.

**Table 4:** Independent samples t-test results of perceived insider status.

Perceived insider status		Workers’ job value status		
		0	1	t-test
Employment form	0	M_1_ = 3.30, SD_1_ = 0.62	M_2_ = 2.12, SD_2_ = 0.69	*t*_12_(145) = −10.90^**^
	1	M_3_ = 4.24, SD_3_ = 0.39	M_4_ = 4.40, SD_4_ = 0.45	*t*_34_(277) = 3.24^**^
	*t*-test	^u^*t*_13_(108) = 12.27^**^	^u^*t*_24_(101) = 24.33^**^	^u^*t*_14_(132) = 13.48^**^, ^u^*t*_23_(85) = 23.75^**^

*N* = 426 (*n*_1_ = 79, *n*_2_ = 68, *n*_3_ = 164, *n*_4_ = 115). SD, standard deviation. For employment form, 0 = outsourced, 1 = standard. For workers’ job value status, 0 = peripheral-status, 1 = core-status.^u^Unequal variance *t*-test (the results of Levene’s test rejected the null hypothesis of variance equality). ^*^p < 0.05, ^**^p < 0.01.

**Table 5:** Independent samples t-test results of job performance.

Job performance		Workers’ job value status		
		0	1	t-test
Employment form	0	M_1_ = 3.63, SD_1_ = 0.45	M_2_ = 2.71, SD_2_ = 0.74	^u^*t*_12_(107) = −8.90^**^
	1	M_3_ = 4.33, SD_3_ = 0.42	M_4_ = 4.46, SD_4_ = 0.41	*t*_34_(277) = 2.54^*^
	*t*-test	*t*_13_(241) = 11.94^**^	^u^*t*_24_(91) = 17.88^**^	*t*_14_(192) = 13.36^**^, ^u^*t*_23_(85) = 16.91^**^

*N* = 426 (*n*_1_ = 79, *n*_2_ = 68, *n*_3_ = 164, *n*_4_ = 115). SD, standard deviation. For employment form, 0 = outsourced, 1 = standard. For workers’ job value status, 0 = peripheral-status, 1 = core-status.^u^Unequal variance *t*-test (the results of Levene’s test rejected the null hypothesis of variance equality). ^*^p < 0.05, ^**^p < 0.01.

### 4.4. Qualitative evidence of the quantitative study

#### 4.4.1. Data collection

To substantiate our quantitative study, we performed an inductive content analysis following Mayring (2014) to identify how employment forms and employees’ job values influence workers’ PIS and job performance. To this end, we asked the different groups questions related to the research findings of the earlier quantitative study. To capture views on these questions, we took the opportunity provided in another large project on the business ecosystem to individually interview (face-to-face) eight standard workers, ten outsourced workers, eight employee supervisors, and five HR workers from September to December 2022. The samples were chosen from the same shipbuilding firms as the quantitative study and all interviewees did not participate in the survey investigation in the quantitative phase. To delve into how workers’ self-categorizations of their relationship with the organization influenced their work experience, we asked the standard and outsourced workers to assess their feelings of being included in the organization. We also probed the PIS of supervisors and HR workers regarding different groups. We then explored how the relationship between employment forms and employees’ PIS varied by employees’ relative value to the organization by asking workers whether they were satisfied with their employment form according to their job positions. In addition, we asked for supervisors’ and HR workers’ observations and thoughts on the matching of employment forms and employees’ relative job value, and the impacts thereof.

#### 4.4.2. Data coding

Each interview lasted about an hour and was tape-recorded and transcribed verbatim. A total of 275 pages of transcribed text was yielded from 31 interviews, of which 67 single-spaced pages (on average, 16 paragraphs and 2,068 words per interview) were used in the analysis conducted for the purposes of this study. The first author trained two coders who were master’s students in management and blind to the hypotheses of the quantitative study. Both coders independently coded all transcripts, compared their codes, and discussed discrepancies. During their independent coding process, they first wrote down reflections and summary notes while reading each transcript, and then combined the open-coded memos of each interview and classified them into key themes. Each step in the analytical process was conducted in face-to-face meetings between the two coders.

#### 4.4.3. Results

Table 6 summarizes the representative observations and quotes from the interviews. The two groups (standard employees vs. outsourced employees) differed in their PIS and job performance along with the influence of employees’ job value diversity. Standard workers, regardless of whether they held core or peripheral positions, considered themselves included in the organization. For example, standard worker ID1003 said: “I am a standard employee with ‘an iron rice bowl’…The company is like my home.” Employees hired in an outsourcing form tended to express feelings of being treated as outsiders and highlighted their intention to withdraw, for example: “I am not sure whether I will stay here in the future” (outsourced worker ID1002). Although core outsourced and peripheral outsourced employees considered themselves more as outsiders, core outsourced employees exhibited more disappointment in their employment status, while peripheral outsourced employees were able to accept their status as outsiders. The views of employees’ supervisors and the HR department supported this point. For example, HR ID3004 stated: “someone (one outsourced worker) said…he feels he is not treated as a member of the company, but as a ‘temporary resource’ that can be thrown away at any time.”

**Table 6:** Coding summary of Interviews with employees, supervisors, and HR workers.

		Standard workers		Outsourced workers	
Interviewee	Interview questions	Observations	Sample quotes	Observations	Sample quotes
*1. PIS*					
Employee	Do you feel like an insider at this organization? Do you feel included in this organization?	More likely to feel as insider	“I have a strong sense of belonging…I have been working here since I graduated from university. My personal growth is synchronized with the growth of the company…I will definitely continue working here until I retire” (standard worker ID1005:5).	More likely to feel as outsider	“I think of myself as working for the service company. I sign the employment contract with the labor service company. My salary is also paid by the labor service company, and my social security is also paid by it” (outsourced worker ID1004:9).
			“Of course, I consider myself an insider. I am a standard employee with ‘an iron rice bowl’. The company is like my home. I met my wife here, got married, and celebrated Chinese New Year with my colleagues here instead of returning to my hometown” (standard worker ID1003:7).		“My feeling is that there is still a gap between us and standard employees…During festivals, the company provides more free benefits to them… Standard workers also took precedence over us in technical training…… I am not sure whether I will stay here in the future” (outsourced worker ID1002:4).
Leader	Views on employees’ PIS	More likely to have higher PIS	“Standard employees are more owner-oriented, specifically, they have a sense of responsibility and care more about the reputation of the company” (leader ID2001:2).	More likely to have lower PIS	“Outsourced employees are more likely to have small groups…although their periods in our company are not very short, they do not think of our company they work for as an organization with a sense of belonging. They are more likely to leave than standard employees” (leader ID2003:4).
HR	Views on employees’ PIS	More likely to have higher PIS	“The company’s services and benefits for standard employees are definitely better than those of outsourced employees, since standard employees can work in the company for a long time, so the investment in their human capital is more worthwhile. Therefore, it is also unquestionable that standard employees feel more like insiders than outsourced employees” (HR ID3002:2).	More likely to have lower PIS	“I have heard outsourced employees’ complain when they go through the resignation procedures, for example, someone said he feels that our company and his leader treats him differently from standard employees, and he feels he is not treated as a member of the company, but as “temporary resource” that can be thrown away at any time” (HR ID3004:5).
*2. Job value*					
Employee	What is your job position? Are you satisfied with the way you are hired?	Both core workers and peripheral workers are satisfied.	“My daily work is technology development, and I signed a fixed contract when I joined the company. Most employees recruited for this position are standard employees…and I feel very stable and secure” (standard worker ID1001:11).	Peripheral workers are satisfied but core workers are not.	“I think the mechanical work I do is more valuable than those assembly line workers’ daily work, but I am also employed in the outsourcing way like them, especially since there are some standard employees with fixed contracts in the same position… I feel unbalanced that the company does not take me seriously…I am still working here now but may go to another company someday” (outsourced worker ID1008:15).
			“My job is mainly to assist technicians to do service. Since I came here many years ago, I got the treatment of standard employees…I can work stably without worrying about leaving here…and I do not have to work too hard to make a living” (standard worker ID1007:10).		“I’m a stevedore, our team is almost outsourced employees… This is normal–we work here to earn money and it is very flexible--if there are no tasks here or if the wage is low, we can change jobs and move to other companies at any time” (outsourced worker ID1003:11).
HR	How employees with different job values are hired? Do you think employees’ job value always matches the way they are hired? Is there any difference in employees’ perception and behavior in the case of matching and mismatching?	High job-value employees are mainly hired in standard employment way, and low job value employees are mainly hired in outsourcing way, but not absolutely--mismatching including employee’s inconsistent perception and behavior exists inevitably.	“Most of the employees in important and core positions are recruited as standard workers. The company retains these ‘key talents’ with fixed contracts and provides them with long-term commitment and guarantee, which is conducive to these core employees continuing to contribute to the development of the company” (HR ID3004:17).	High job value employees are mainly hired in standard employment way, and low job value employees are mainly hired in outsourcing way, but not absolute--mismatching including employee’ s inconsistent perception and behavior exists inevitably.	“Due to the improvement of business operating costs control and flexibility, the proportion of outsourced employees has increased in recent years, regardless of position…The dissatisfaction of outsourced employees in core positions is very prominent–they believe that their “status” is different from standard employees in the same department even in the same position…outsourcing employment is uncertain which means outsourced employees are not recognized by the enterprise, and they may leave at any time actively or passively” (HR ID3002:22).
			“Hiring standard employees or upgrading outsourced employees who work for a long time to standard employees in peripheral positions is one of the ways to motivate employees… After all, giving employees standard status is conducive to increasing their sense of belonging and commitment because they are more likely to think themselves as insiders if they obtain permanent employee status” (HR ID3005:23).		The use of outsourced employees in peripheral positions has become a common practice in the industry, such as some ordinary skilled workers, sales, and service employees, who are in great demand and mobility -- for enterprises, the labor market does not lack workers; and meanwhile for workers, there are also enough companies they can go to work. Therefore, outsourcing employment is in the interests of both sides” (HR ID3003:22).
*3. Job Performance*					
Leader	How employees with different employment ways perform at work?	More likely to have high performance	“It is not surprising that standard employees generally perform better–they have been working in this company for a long time, they see the company as their own home…they perceive themselves as the ‘home owner’ and naturally have a responsibility to behave well to make the home better” (leader ID2001:34).	Relatively have a lower level of performance than standard workers	“Although the administration of outsourced employees is carried out by our company and the agency company together, their salary and social security are all managed by the agency company…Outsourced workers are more likely to complete some certain ‘packaged tasks’, maybe completing this task here today but instantly going to another company tomorrow to complete other tasks…their performance can not be stable and excellent as standard employees” (leader ID2003:25).
HR	How employees with different employment ways perform at work?	Overall core standard workers perform consistently well	“Core standard workers always show outstanding performance and powerful sense of responsibility to their work” (HR ID3003:34).	Performance varies widely within outsourced workers	Those outsourced workers in core positions have the ability and opportunity to make the transition to other companies at any time, unless they can obtain standard status here… and the mismatching between the core value job position and the outsourcing employment greatly dampen employees’ identity and motivation to perform well” (HR ID3001:41).
			“Some peripheral employees are less motivated to work hard after receiving standard employment contracts…they may think that they will not be at risk of dismission even if they behave poorly” (HR ID3005:49).		“Peripheral employees are accustomed to this extensive outsourcing employment way…their job performance may be not outstanding but also not bad” (HR ID3002:31).

In terms of the boundary conditions of job value that shifted employees’ PIS, both core standard and peripheral outsourced employees were satisfied with their status and took workforce employment diversity for granted. For example, standard employee ID1001, an R&D staff, noted: “Most employees recruited for this position are standard employees…and I feel very stable and secure.” Peripheral outsourced employees also took an impartial attitude toward their employment form. According to outsourced worker ID1003, “Our team is almost outsourced employees…we work here to earn money and it is very flexible—If there are no tasks here or if the wage is low, we can change jobs and move to other companies at any time.” In comparison, the opinions of core outsourced and peripheral standard employees varied substantially. When asked if they were satisfied with the way they were hired, core outsourced employees mentioned being more dissatisfied than their standard peers. For example, as per outsourced worker ID1008: “I feel unbalanced that the company does not take me seriously…I am still working here now but may go to another company someday.” However, standard peripheral employee ID1007, who has been with the company for many years, said: “I can work stably without worrying about leaving here…and I do not have to work too hard to make a living.” This reflects less effort made to behave well when peripheral workers acquire standard status.

Perspectives from HR personnel and supervisors further supported the findings that job value status influenced the relationship between employment forms (standard vs. outsourcing) and job performance *via* PIS. HR personnel expressed support for the views that high job value employees should be mainly hired in a standard employment form, while low job value employees should be hired in an outsourcing form. However, this was not the case in reality: There was evidence of mismatching such as employees’ inconsistent perception and behavior. For example, HR ID3004 noted that companies retained core standard workers as “key talents” with fixed contracts and guaranteed to promote them to contribute to the development of the company. HR ID3005 added that upgrading employees to standard status in peripheral positions was also a way to motivate employees to increase their sense of belonging and commitment. Meanwhile, “the use of outsourced employees in peripheral positions has become a common practice…outsourcing employment is in the interests of both sides (enterprises and workers)” (HR ID3003). However, “the dissatisfaction of outsourced employees in core positions is very prominent…outsourcing employment is uncertain, which means outsourced employees are not recognized by the enterprise” (HR ID3002).This reflects that core outsourced employees were more sensitive to an imbalanced form of employment. Some supervisors suggested that the performance of outsourced workers may not be as stable and outstanding as that of standard employees (leader ID2003), who “perceive themselves as the ‘home owner’ and naturally have a responsibility to behave well to make the home better” (leader ID2001). Moreover, some HR personnel (e.g., HR ID3001) emphasized that “the mismatching between the core value job position and outsourcing employment greatly dampen employees’ identity and motivation to perform well.” Overall, these perspectives were aligned with what is characterized as the human resource architecture model and corroborate our empirical findings.

## 5. Discussion

Most research has focused on the effects of flexible employment on changes in work arrangements (e.g., short-time work, paid short break, flexible location and hours, and financial consequences) for workers in regular employment relationships. However, there has been less discussion on the experiences of employees in flexible employment relationships. Those employees are neither officially laid off, nor offered insurance or other compensation depending on country-specific labor laws. In this study, we sought to answer the fundamental question of when and how outsourced employment has internal discrepancies and how outsourced employees experience and behave differently compared to standard employees. Regarding this question, the present study examined the relationship between workers’ employment forms (standard vs. outsourced) and their job performance, with PIS as a mediator that is further moderated by employees’ job value status. Our analyses support the proposed hypotheses that compared with outsourced employees, standard employees exhibit higher job performance, and PIS plays a mediating role between employment forms (standard vs. outsourced) and their job performance. Furthermore, employees’ job value status (core vs. peripheral) moderates the strength of the mediated relationship between employment form (standard vs. outsourced) and job performance *via* PIS, such that the mediated relationship is stronger among the core-status workers than among the peripheral-status workers. Our additional qualitative study substantiates the validity of these results by finding agreement across various research strategies (Turner et al., 2017).

### 5.1. Theoretical implications

Our findings offer several theoretical contributions to the existing literature on outsourced workers and nonstandard employment in general. First, our work responds to Ashford et al. (2007) as well as Spreitzer et al.’s (2017) call “to bring the study of nonstandard work more to the center stage of particularly micro-OB.” By providing new empirical evidence on the difference between outsourced and standard employees’ work experience and quality, this study deepens the current understanding of outsourced employment. On the one hand, our findings are consistent with previous theoretical and empirical assertions that nonstandard employees are disadvantaged in terms of performance. On the other hand, following Lapalme et al.’s (2009) discussion and empirical evidence that an insider perception not only exists, but is also vital to outsourced workers’ work experience, we point out the extent to which employees perceive themselves as organizational insiders as a new mechanism that explains how the disadvantage might happen. Moreover, our work extends existing knowledge on the psychological underpinnings of nonstandard employees’ work experience, responding to De Cieri et al.’s (2022) call to explore the effect of flexible employment on employees’ attitudes. While alternatives to the archetypal model of traditional standard employment are prevalent and wide-ranging, most management science notions about nonstandard employment take a more macro rather than individual approach. This includes the worker contractualization issue, employment relations, institutional changes, and workforce fragmentation (Hipp et al., 2015). Why and how nonstandard workers exhibit different attitudes and behaviors is still lacking scholarly investigation. By focusing on subjective insider perception, this study enriches this stream of research and complements our understanding of objective organizational inclusion.

Second, by introducing a balanced employee-organization architecture model, we offer a new lens by which to understand the triggers that drive the difference in job quality between outsourced employees and their colleagues in a standard employment form. As such, we respond to the call regarding the impact of outsourcing on the role and effect of the HR function and employment relations (Zhao et al., 2021). Specifically, we proposed core-outsourced and peripheral-standard groups as two unbalanced groups, and distinguished them from the other two balanced groups, namely the core-standard and peripheral-outsourced groups. We found that employees in the unbalanced core-outsourced group exhibited significantly lower PIS and worse performance than both core-standard and peripheral-outsourced employees. Employees in the other unbalanced peripheral-standard groups also showed significantly higher PIS and better performance than peripheral-outsourced employees, but lower PIS and worse performance than core-standard employees. In other words, while both unbalanced groups are disadvantaged in terms of their psychological closeness with the organization and their performance, core employees are more sensitive to whether their employment form “matches” their job value status as opposed to peripheral employees. For employers, an under-investment (core-outsourced) might lead to a lack of work motivation, an over-investment (peripheral-standard) could be a waste of resources, and a balanced investment (core-standard and peripheral-outsourced) is more favorable. This further confirms earlier scholarly assertions that organizations should adopt appropriate employment forms for employees in different positions to increase business flexibility and stability (Tsui et al., 1997; Lepak and Snell, 2002; Signoretti et al., 2022).

### 5.2. Practical implications

Our findings offer several practical implications for employers, managers, and nonstandard workers. For employers, we suggest that where to use nonstandard workers is critical for overall work effectiveness. Organizations should apply differential HRM systems in different contexts (Liu et al., 2021). When the positions are of high value to an organization, a nonstandard workforce should be used with caution. If using nonstandard workers is unavoidable due to certain considerations (e.g., hiring quota is constrained by local policy), employers should take proactive actions to improve their employment and work experience, such as arranging regular communications with sympathy, supplying training resources for their future career development, and offering opportunities to transfer to standard employment. Besides, HR should implement management in the process of socialization targeting different types of employees, such as customized training for newcomers according to workers’ employment forms and job value status, to make new staff clearer about their relationships with the organization and reduce the social comparison between different groups (Manuti et al., 2017).

Managers, especially those middle managers responding to managing both standard and nonstandard employees in one work unit or group, should pay attention to their management of both groups in their daily work. For example, our findings on the mediating role of PIS between employment forms and job performance suggest that managers could take initiatives to cultivate subordinates’ identity recognition as insiders of the organization (Dai and Chen, 2015) and promote a securer work atmosphere. Managers should also be alert to employees’ emotional cues so that they can take precautionary actions rather than implement remedies afterward.

For workers, our results suggest that both insider perception and job value status are critical drivers of their performance. As such, workers should take a more comprehensive approach to viewing their jobs instead of simplifying all nonstandard jobs as “bad jobs.” Rather than reinforcing the disadvantages, outsourced employees could focus more on the value improvement of their work and become proactive in reshaping and enlarging the boundaries of their job (e.g., crafting) to gain access to opportunities to promote or transfer to standard employment. Moreover, individuals should also recognize that even when they are in a nonstandard employment situation, they should actively blend in with teams, work units, and the organization they belong to. By embracing the challenges brought about by the employment form, they can gain better and improved job performance and benefit their career development.

### 5.3. Limitations and future research directions

Like all research, this study has certain limitations. First, despite outsourcing being a major form of employment in the nonstandard family, focusing thereon constrains the generalizability of our findings to other nonstandard forms such as part-time, on-call, and temporary employment. For example, Haines et al. (2018) argued that a configuration of various types of part-time employment is necessary to understand part-time workers’ work experience and outcomes. In the future, researchers could integrate other types of nonstandard employment forms into their studies and explore the nuances within other types of nonstandard employment forms and differences from standard employment.

Second, although multiple efforts were made to rule out potential common method bias, including multi-source and time-delayed data collection, proxying objective job information rather than using subjective assessment, and rigorous supplementary analyses as Podsakoff et al. (2003, 2012) suggested, causality cannot be determined based on the findings of the current study. We encourage future research to adopt experimental or longitudinal designs to better test the causality between employment forms, psychological experience, and resulting performance.

## 6. Conclusion

This study focused on the new trend of flexible employment and investigated attitudinal and behavioral outcomes at the employee level of outsourcing employment. Drawing on status characteristics and social categorization theories, we proposed the main negative effects of outsourcing on employees’ PIS and then job performance, as well as the contingency effect of employees’ job value status. The full model was empirically supported. The study not only depicts new avenues for future research on flexible employment, but also suggests to employers how to manage employee diversity based on both subjective and objective status.

## Data availability statement

The raw data supporting the conclusions of this article will be made available by the authors, without undue reservation.

## Ethics statement

The studies involving human participants were reviewed and approved by the Renmin University of China. Informed consent was obtained from all individual participants included in the study. The patients/participants provided their written informed consent to participate in this study.

## Author contributions

JY, WS and EC proposed the research idea and designed the work. EC and BL improved the theorizing. JY, ZZ, and ZY collected, screened, and analyzed the articles together. JY, EC, ZZ, ZY, and JT revised the draft. All authors contributed to the article and approved the submitted version.

## Funding

This work was supported by the Young Scientists Fund of the Ministry of Education of Humanities and Social Sciences Project in China (Grant no. 21YJC630010).

## Conflict of interest

The authors declare that the research was conducted in the absence of any commercial or financial relationships that could be construed as a potential conflict of interest.

## Publisher’s note

All claims expressed in this article are solely those of the authors and do not necessarily represent those of their affiliated organizations, or those of the publisher, the editors and the reviewers. Any product that may be evaluated in this article, or claim that may be made by its manufacturer, is not guaranteed or endorsed by the publisher.
